# A Regional Pooling Intervention in a High-Throughput COVID-19 Diagnostic Laboratory to Enhance Throughput, Save Resources and Time Over a Period of 6 Months

**DOI:** 10.3389/fmicb.2022.858555

**Published:** 2022-06-09

**Authors:** Prerna Mandhan, Mansi Sharma, Sushmita Pandey, Neha Chandel, Nidhi Chourasia, Amit Moun, Divyani Sharma, Rubee Sukar, Niyati Singh, Shubhangi Mathur, Aarti Kotnala, Neetu Negi, Ashish Gupta, Anuj Kumar, R. Suresh Kumar, Pramod Kumar, Shalini Singh

**Affiliations:** ICMR-National Institute of Cancer Prevention and Research, Noida, India

**Keywords:** COVID-19, SARS-CoV-2, pooling interventions, high-throughput diagnosis, RT-qPCR–real-time quantitative polymerase chain reaction

## Abstract

An effective and rapid diagnosis has great importance in tackling the ongoing COVID-19 pandemic through isolation of the infected individuals to curb the transmission and initiation of specialized treatment for the disease. It has been proven that enhanced testing capacities contribute to efficiently curbing SARS-CoV-2 transmission during the initial phases of the outbreaks. RT-qPCR is considered a gold standard for the diagnosis of COVID-19. However, in resource-limited countries expenses for molecular diagnosis limits the diagnostic capacities. Here, we present interventions of two pooling strategies as 5 sample pooling (P-5) and 10 sample pooling (P-10) in a high-throughput COVID-19 diagnostic laboratory to enhance throughput and save resources and time over a period of 6 months. The diagnostic capacity was scaled-up 2.15-folds in P-5 and 1.8-fold in P-10, reagents (toward RNA extraction and RT-qPCR) were preserved at 75.24% in P-5 and 86.21% in P-10, and time saved was 6,290.93 h in P-5 and 3147.3 h in P-10.

## Introduction

The coronavirus disease 2019 (abbreviated “COVID-19”) first appeared in China in 2019 and was declared a pandemic on 11 March 2020 (Zhu et al., 2020). Still, the disease represents a global challenge due to the high transmissibility of the virus (SARS-CoV-2) as millions of cases are reported from several countries across the globe. Till 21 October 2021, the aggregate number of cases reported globally exceeded 241 million and the number of global deaths was approximate 4.9 million according to the WHO (Phelan et al., 2020; World Health Organization [WHO], 2021). To tackle the COVID-19 pandemic, especially different waves and the emergence of the variants, effective diagnosis is key to combat the disease (Vandenberg et al., 2021).

Despite the various interventions like physical distancing, face masking, vaccines, and antivirals, effective and rapid diagnosis has great importance in tackling the pandemic through isolation of the infected individuals to curb the transmission and facilitation of the targeted therapy for COVID-19 (Zamora-Ledezma et al., 2020). It has been observed that countries with effective testing capacities efficiently curb transmission of the virus during the initial phase of an outbreak during the ongoing pandemic (Wang et al., 2020). Molecular tests are proving their potential as better diagnostic techniques which are being used in the testing and evaluation for a larger sample size (Beal et al., 2016; Chauhan et al., 2020, 2021; Singh et al., 2020). Reverse Transcription Quantitative Polymerase Chain Reaction (RT-qPCR) is a gold standard for COVID-19 diagnosis due to its high sensitivity and accuracy but the expenses limit its application, particularly in resource-limited countries. Considering the large population base, cost-effective and rapid tests are required on an urgent basis to reduce the spread of the SARS-CoV-2 (Kumar et al., 2020; Tahamtan and Ardebili, 2020).

Pool testing (also referred to as pooling) is a testing strategy involving the mixing of the same type of specimens to perform one laboratory test using the mixed pool of the specimens to detect the viral target. The pool specimens tested positive, need retesting of each sample (individually) from the respective pool to identify individual positives. If a pool test results negative, all the specimens from the respective pool are declared negative (Daniel et al., 2021). It has been proposed as a strategy to maximize the number of experiments conducted, saving diagnostic resources, reagents, reducing time to declare negative results, greater throughput at lower costs, and overall increasing the diagnostic capacity (Lagopati et al., 2020). The high diagnostic capacity would cover a larger sample size including asymptomatic, close contacts, and epidemiological surveillance. Thus, enable timely curbing of the infection and prevents further spread in the community. Pooling can be done at the level of the clinical specimen (before RNA extraction) or after RNA extraction (Lohse et al., 2020).

Pool testing strategy has been used previously in the diagnosis of other infectious diseases and is also recommended in the case of COVID-19 for keeping a tab on disease transmission. The concept of pool testing was proposed by Dorfman in 1923. This is helpful in screening samples for larger populations in case of both asymptomatic as well symptomatic cases (Mutesa et al., 2021). Though this is quite useful with limitations of generation false-negative results as pooling leads to dilution of samples. Hence the sample pool consists of 5 or 10 individual samples, followed by individual re-testing (deconvoluted) only of pools that tested positive (Garg J. et al., 2021).

## Methodology

Nasopharyngeal swabs (NPS)/Oropharyngeal swabs (OPS) were collected from the patients and transported in viral transport medium (VTM) to high-throughput viral diagnostic laboratory, ICMR-NICPR, Noida. The VTMs were transported from different collection centers or hospitals in triple-layered packaging with the maintenance of the cold chain. Once the samples were received in the laboratory they were immediately processed for the diagnosis and in case of any delay the samples were stored in 4°C cabinets in the laboratory until processed. The samples were heat-inactivated at 56°C for 30 min, after inactivation, a 180 μl sample was aliquoted in each well (96 deep well plate) and immediately processed for RNA extraction by automated extraction machines MGISP-960 using MGIEasy Magnetic Beads Virus DNA/RNA Extraction Kit (MGI, 1000020471). To avoid cross-contamination while high-throughput diagnosis, all the steps involving sample opening or reagent prorations starting from sample sorting, aliquoting, RNA extraction, reagents predation, template addition, and PCR amplification was done in dedicated areas equipped with biosafety cabinets.

Pool testing was first started in samples received from different districts (Amroha, Baghpat, Bijnor, Bulandsher, Hapur, Kasganj, Mathura, Moradabad, Rampur, Shambhal, and Shamli) of western Uttar Pradesh (UP) from 6 October 2020 based on the low prevalence of COVID-19 as observed in the previous week. In mid-November 2020 individual testing of the UP, samples were performed due to a rise in positivity rate. Following the reduction in positivity rate, pool testing was again initiated. At the end of January 2021, we noticed a further reduction in the positivity rate in UP and Delhi samples then initiated the 10-sample pool testing in samples received from Delhi and UP.

### Pooling of 5 (P-5) Samples

Pooling of samples for the diagnosis of COVID-19 was started as per the guidelines of the Indian Council of Medical Research (ICMR) (Garg J. et al., 2021) with slight modification in the P-10 strategy. The positivity rate in pooling was calculated as the number of positive pools tests divided by the total number of pool tests, and the outcome was multiplied by 100. Five samples of NPS/OPS (P-5) were pooled before the RNA extraction P-5 strategy was used from 5 October 2020 to 16 January 2021. A 100 μl aliquot of each sample was dispensed in a deep well plate from the VTM vial. Each well of the deep-well plate (stock plate of P-5) contained 500 μl (5 samples × 100 μl). After pooling of samples in the stock deep well plate, samples were gently mixed using multichannel pipettes by inserting the micro-tips up to the bottom of the deep-well and repeated pipetting and 180 μl of each pool was transferred to a new plate (working -plate) for RNA extraction. In the P-5 strategy, 470 samples (94 × 5: 470; negative control; positive control) were tested in a single run. A total of 76,068 pools (76,068 × 5: 380,340 specimens) have been tested from 5 October to 16 January using the P-5 strategy.

### Pooling of 10 (P-10) Samples

Ten samples of NPS/OPS were pooled before the RNA extraction. The P-10 strategy was used from 17 January 2020 to 31 March 2021. A 100 μl aliquot of each sample was dispensed in a deep well plate from the VTM vial. Each well of the deep well plate (stock plate of P-10) contained 1,000 μl (10 samples × 100 μl). After pooling of samples in a stock deep well plate, samples were mixed using multichannel pipettes and 180 μl of each pool was transferred to a new plate (working-plate) for RNA extraction. In the P-10 strategy, 940 samples (94 × 10: 940; negative control; positive control) can be tested in a single run. A total of 43,146 Pools (43,146 × 10: 431,460 specimens) have been tested from 17 January to 31 March 2021.

### RNA Extraction

All extraction protocols were followed as per RNA extraction kit instructions. In brief, a total of 180 μl sample was added into 96-deep well plate followed by the addition of a freshly prepared mixture of lysis and binding buffer which includes 15,680 μl MLB, 19,600 μl ethanol, 98 μl enhancer buffer, 1,470 μl proteinase K, and magnetic beads, respectively. Buffer mixture was freshly prepared and used within 30 min. The sample plate, reagents deep-well plate, and filter-tip boxes were kept at their respective positions as per the extraction kit protocol. Once the RNA was extracted, immediately processed for the RT-qPCR, and in case of any delay, the samples were stored in 4°C cabinets for a few hours or stored at –20°C for 1–2 days until processed and one aliquot of the RNA was stored –80°C.

### Reverse Transcription Quantitative Polymerase Chain Reaction

RT-qPCR was performed by using TaqMan assay chemistry on the Bioer platform (Line gene 9600 series) by using distinct diagnostic kits such as BGI (Beijing Genomics Institute, Shenzhen, China), GENES2ME (Genes2Me Pvt. Ltd., Gurugram, Haryana, India) COVISURE (Genetix Biotech Asia Pvt. Ltd., New Delhi, India), Q LINE and COVIWOK approved for the COVID-19 diagnosis by ICMR/WHO/FDA have been used in the testing (Table 1). Amplification of target sequence in SARS-CoV-2 RNA confirmed the presence of virus in the sample. Beta-actin and Rnase P are used as a housekeeping genes for internal control.

**TABLE 1:** Details of the Kits used for RT-qPCR setup in diagnosis of the SARS-CoV-2.

Kits used	Company name	Viral target gene	Viral target CT value cut off ≤ (as per ICMR guidelines)	Cut off used in P-5	Cut off used in P-10
COVISURE Genetix	Genetix Biotech Asia Pvt. Ltd., New Delhi, India	E GENE	35	38	39
		RdRp			
		Internal Control			
BGI Genomics	Beijing Genomics Institute, Shenzhen, China	ORF1ab	35	38	39
		Internal Control			
Q LINE	Q LINE, India	N GENE	35	38	39
		ORF1ab			
		Internal Control			
GENES2ME viral detect II	Genes2me Pvt. Ltd., Gurugram, Haryana, India	E GENE	35	38	39
		RdRp			
		Internal Control			
COVIWOK	SNP Biotechnology R&D Ltd., Hacettepe Technopolis, Ankara/Turkey	RdRp	35	38	39
					

Pools tested negative in RT-qPCR were reported as negative for all individual samples whereas retesting of all individual samples from the presumptive positive pool were done to confirm the positive result in the individual sample.

### Interpretation of Reverse Transcription Quantitative Polymerase Chain Reaction Results

During amplification, the fluorescence emission signals correspond to amplicons of viral targets detected in real-time and the fluorescence emission data is plotted against the *in vitro* replication cycles. The cycle threshold (Ct) or threshold cycle value is the cycle number at which the fluorescence generated within a reaction crosses the fluorescence threshold, a fluorescent signal significantly above the background fluorescence. The Ct for individual testing was considered as per mentioned in the kit used. Ct values are inversely proportional to the viral load in the sample. A higher Ct value indicates a lower viral load while a lower Ct value indicates a high viral load in the sample. In the pooling strategy, there are chances of missing the low positive cases as the viral load of low positive cases is diluted with an increased volume of negative specimens in pools. To consider borderline positive cases, the Ct value for viral targets in P-5 and P-10 samples testing exceeded corresponding to the individual sample testing by +3 and +4 in the cut-off to declare a pool positive. For tenfold dilution of a template and 100% efficiency of RT-qPCR, the slope should be + 3.33. However, at 90% efficiency, the slope would be + 3.6. We have used + 4 as a complete number. For fivefold dilution of a template and 100% efficiency of RT-qPCR, the slope should be + 2.33. However, at 90% efficiency, the slope would be +2.6. We have used +3 (for 2.33–2.6) and + 4 (3.33–3.6) as complete numbers in P-5 and P-10 strategies, respectively. This was only considered amplification of both the genes (target and internal) result is considered as positive, even if no amplification of screening gene (E Gene/N Gene) but the presence of a confirmatory gene (RdRp/ORF 1ab) also concluded as positive and result considered as negative if amplification observed in internal control only. The absence of amplification of the target and internal gene were defined as an invalid reaction and considered for repeat-testing.

Most analyses were conducted in Microsoft Excel. The confidence intervals were calculated using: https://www.socscistatistics.com/confidenceinterval/default4.aspx. The confidence interval was calculated using *t*-test and two means of two groups to generate an interval estimate of the difference between the groups.

## Results

### P-5 Testing Strategy

P-5 testing strategy was performed in OPS/NPS samples for diagnosis of COVID-19 at a high high-throughput laboratory over a 15-week period from 5 October 2020 to 16 January 2021. A total of 380,340 samples were tested using 76,068 pools (P-5). Corresponding to these, 4.76% of pools were flagged as positive based on screening or confirmatory targets of the viral genome and the respective specimens were subjected to individual testing. In this strategy, 1.08% of individual specimens were diagnosed as positive for SARS-CoV-2. While in deconvolution, the flagged positive pools contain a single positive specimen (51.44%), multiple positive (23.16%), none positive (22.9%), and resample (1.99%).

**FIGURE 1:**
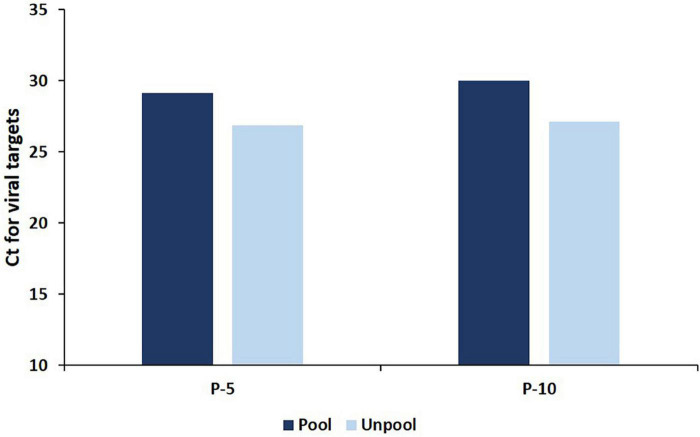
The average Ct for viral targets in deconvoluted (unpool) samples and in pools (P-5 and P-10 strategy) which resulted in only one positive specimen in deconvolution.

An in-depth analysis of the Ct value distribution was performed to check the pooling effect in pools that contain single positive samples with low viral loads. The average Ct for viral targets in the P-5 strategy was observed as 29.16 and for deconvoluted samples, it was 26.89 and showing a mean loss of 2.26 Ct (95%CI, 2.08 2.46) value for the viral target gene (Figure 1) in the pools which resulted in only one-positive samples in deconvolution. Samples (44.67%) were showed Ct ≥ 30 in the P-5 strategy, with one positive sample in each pool. The average Ct value of P-5 samples and unpool was 33.71 and 31.23, respectively and a mean loss of 2.48 Ct (95%CI, 2.32–2.63) value was observed. Whereas 55.33% samples (*n* = 581) were showed Ct < 30 and 44.6% samples (*n* = 469) showed Ct ≥ 30 in the P-5 strategy, with one positive sample in each pool. The average Ct value of P-5 samples and unpool was 25.48 and 23.38, respectively and a mean loss of 2.09 Ct (95%CI, 1.93 2.25) Ct for viral target genes was observed.

**FIGURE 2:**
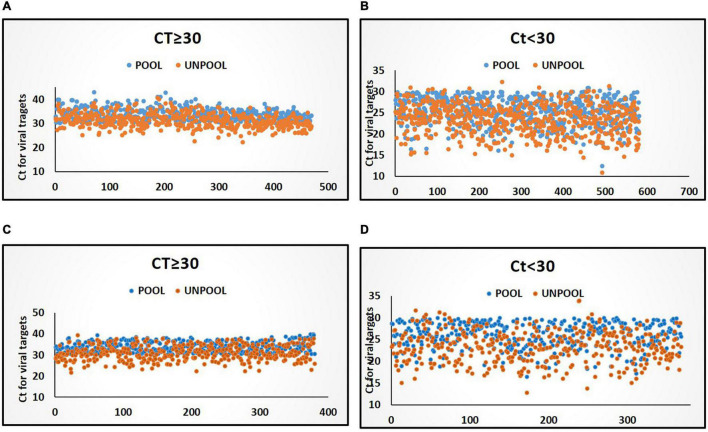
Distribution of Ct values for viral target in pools and deconvoluted samples with only one positive sample in deconvolution. **(A)** Distribution of Ct values (≥30) in the P-5 testing strategy. **(B)** Distribution of Ct values in (<30) in P-5 testing strategy. **(C)** Distribution of Ct values (≥30) in P-10 testing strategy. **(D)** Distribution of Ct values in (<30) in P-10 testing strategy. Blue and orange color markers indicate Ct of viral targets in pools and in deconvoluted samples, respectively.

The laboratory positivity rate was higher than the regional positivity and P-5 strategy was adopted in samples received from the fewer diseases prevalence areas (Districts of Uttar Pradesh). The average lab positivity rate was around 5.40%, but in specific regions (districts of Uttar Pradesh) the disease prevalence was less, which led to the implementation of the P-5 strategy on region-wise. The regional positivity in the pooling of the P-5 strategy for SARS CoV-2 ranged between 0.14 and 4.04% with an average of 1.24% (Figure 2).

During the span of 4 weeks, i.e., from 12 November to 8 December the prevalence of SARS-CoV-2 was high and the laboratory positivity rate jumped up to 13.34% which forced to drop in the pooling strategy. The maximum lab positivity rate was observed at 19.32% in the 3rd and 4th weeks of November. After the 2nd week of December, there was a dip in the positivity rate from 16 to 6%, and the P-5 testing strategy was resumed. From 15th December onward, the laboratory positivity was dropped below 1% in all the regions of samples collection which led to implementing pooling in all the samples received from Delhi and UP in the laboratory, thus both laboratory positivity and regional positivity became identical. From 17 January onward the positive rate of pooling was beyond dropped <0.5% and the P-10 (pooling of 10 samples) strategy was initiated.

### P-10 Testing Strategy

A total of 431,460 samples or 43,160 pools (P-10) were screened from 17 January 2021 to 31 March 2021, corresponding to these 3.49% pools were flagged as positive based on screening or confirmatory targets of the viral genome. After deconvolution, 0.46% of individual specimens were diagnosed as positive for SARS-CoV-2. The flagged positive pools contain single positive specimens (51.43%), multiple positive specimens (29.16%), and none positive specimens (19.4).

An in-depth analysis of the Ct value distribution was performed to check the pooling effect in pools. The average Ct for viral targets in the P-10 testing strategy was observed as 29.98 and for deconvoluted samples, it was 27.14 and showed a mean loss of 2.84 Ct for the viral target gene (Figure 1) in the pools which resulted in only one-positive samples in deconvolution. 49.19% samples were showed Ct < 30 in the P-10 strategy, with one positive sample in each pool The average Ct value of pool and unpool was observed as 25.98 and 23.24, respectively, and a mean loss of 2.78 Ct) for viral target genes was observed. Whereas 48.57% samples (*n* = 376) were showed Ct < 30 and 51.42% samples (*n* = 398) showed Ct ≥ 30 in P-5 strategy, with one positive sample in each pool. The average Ct value of 10 sample pools and unpool was 34.12 and 31.06, respectively and a mean loss of 3.05 Ct for viral target genes was observed. The regional (also laboratory) positivity of the P-10 strategy for SARS CoV-2 ranged between 0.03 and 2.15% with an average of 0.43%.

### Consumables and Resource Preservation

In the study, if all the specimens were tested without pooling, 811,800 reactions would have been consumed for a similar number of specimens, i.e., 811,800 from 5 October 2020 to 31 March 2021. Moreover, 8,637 batches for RNA extraction and RT-qPCR (94 samples per plate) would have been consumed.

In P-5 testing strategy, among 380,360 samples, 98,266 reactions were consumed which include 76,068 pool-reactions + 18,120 de-convoluted reactions (3,624 positive pools × 5 = 18,120). In this strategy, 1,002 batches of both RNA extraction and RT-qPCR were performed for 380,340 specimens which saved 75.24% batches as compared to individual testing.

Whereas in the P-10 strategy, 556 batches of both RNA extraction and RT-qPCR were consumed for 59,497 reactions (431,460 specimens). Therefore, only 13.79% of consumables for RT-qPCR and RNA extraction were consumed, while consumables for 371,963 reactions were saved, resulting in a huge quantity (86.21%) of reagents preservation as compared to individual testing of the specimen.

### Pooling as a Time-Saving Strategy

**FIGURE 3:**
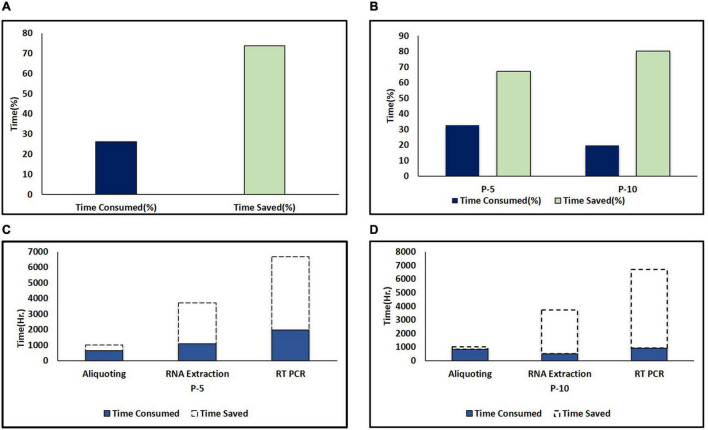
Time saved in pooling strategies. **(A)** Percent time saved during pooling strategies (combined of P-5 and P-10). The dark color column represents time consumed and the light color column represents time saved. **(B)** Comparison of percent time saved in P-5 and P-10 testing strategies. **(C)** Time saved in hours during different areas of testing including aliquoting, RNA extraction, and PCR during the P-5 testing strategy. **(D)** Time saved in hours during different areas of testing including aliquoting, RNA extraction and PCR during P-10 testing strategy. The dark color column and area under the dash line indicate the consumed and saved time in different areas of the testing protocol due to pooling strategies.

Pooling strategy not only played a major role in resource-saving but also it was observed that with P-5 and P-10 pooling strategies, more time was saved when compared to individual testing (Figure 3A). However, more time was taken for pool preparation as compared to aliquoting individual samples. Each plate of the individual specimen (94 specimens) took an average of 15 min, while the P-5 strategy on average took 45 min/plate (470 specimens), and moreover P-10 strategy took an extra 1 h and lead to 115 min/plate (940 specimens) of aliquoting, 30 and 100 extra minutes were consumed in P-5 and P-10 strategies, respectively, in comparison to individual sample diagnosis for aliquoting in a single batch. The time taken for RNA extraction on an average was 55 min, during individual testing of samples only 752 samples were processed with 4 machines on the run while using the P-5 strategy 3,760 samples were processed with 4 machines on the run, and similarly with the P-10 strategy 7,520 could be processed in the same amount of time but when RT-qPCR results were taken into account, the average run time for a single PCR-Plate was around 99 min, while earlier during individual testing (disease prevalence high) and we were able to test 94 samples in a span of 99 min, but with the adoption of pooling strategies in the same period of time, we were able to test 470 sample (P-5 strategy) and 940 samples (P-10 strategy) within the given 99 min, hence increasing our efficiency by a staggering 400 and 900%, respectively (Figure 3B).

With a lab testing capacity of 6,000 samples/day, during individual sample testing due to the high prevalence of SARS COV-2, 3,181 samples/day with all the RNA extraction machines and PCR machine on the run, due to this delay in result updating was also observed, consumption of resources such as testing kits, manpower, and load on necessary machinery increased. An average of 6,850 samples/day were diagnosed using the P-5 testing strategy during the study period which showed a scale-up of 2.15 times diagnostic efficiency as compared to individual testing (3,699 samples/day), (i.e., 115.34% more samples) and hence crossing our testing capacity by 850 samples. Thereby we saved time, valuable resources, manpower, and this reduced load on necessary machinery as well. We were also able to deliver results on time.

A further dip in the positivity rate was observed and the P-10 strategy was opted which further increased the diagnostic efficiency to 1.8 times of individual testing and tested 5,741 samples/day (i.e., 80.47% more samples) (Figure 3C). However, during the P-10 strategy samples received in the laboratory were also less due to the lowest incidence of the disease during the study period.

However, for RNA extraction and PCR in P-5 and P-10 strategies, 2,790.33 and 5,022.6 h and 3,205.59 and 5,077.05 h were saved respectively, because more number of samples were tested at the same time (Figure 3D). Hence, overall, in the P-5 and P-10 strategies, we were able to save 377,456 min (6,290.93 h) and 188,838 min (3147.3 h), respectively, when compared with individual testing.

**FIGURE 4:**
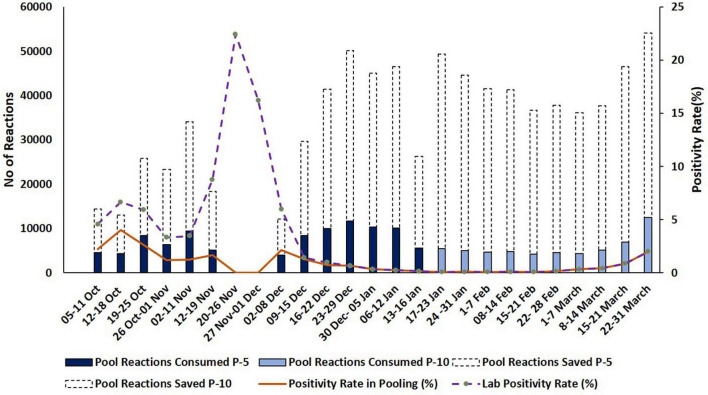
Representation of resources consumed and saved during pooling strategies. Dash type trend line and dark color trend line indicate the percentage of lab positivity and the positivity rate in pooling during the testing period, respectively. *Y*-axis indicates reactions saved in numbers and positivity rate in percentage.

Our data suggest that the P-5 strategy is very effective and time-saving. When disease prevalence is low P-10 strategy has proved to be a game-changer in saving time, money, testing kits, manpower, and other resources. As well as effectively identifying the infected SARS-CoV-2 individuals in the population rapidly (Figure 4).

## Discussion

In this study, we demonstrate that pooling is a feasible diagnostic strategy for SARS-CoV-2 detection in clinical samples, with which comparative sparing of nucleic acid extraction kits and PCR reagents could be accomplished and test throughput could be significantly increased. RT-qPCR is considered the gold standard technique for the detection of SARS-CoV-2 due to its high specificity, sensitivity, and reliability. Both the P-5 and P-10 strategies were effective in bulk testing of COVID-19 in comparison with individual testing without compromising the quality of the outcome of the test. In particular, pooling strategies are well adopted in the mass screening of a population. Here, it has been demonstrated that a regional pooling approach facilitated the high-throughput diagnosis of COVID-19 in the laboratory.

Pools of 5 and 10 samples could be processed with modified RT-qPCR workflows in the context of a very busy diagnostic laboratory. As various studies show, test sensitivity is inversely proportional to testing efficiency depending on pool size.

However, there was a surge of COVID-19 cases in India during November 2020 which resulted in a high laboratory positivity (15.08%) from mid-November to 30 November. We have abolished the pooling strategy during this period as positivity was higher in all sampling areas, P-5 pooling strategy again started on 2 December following a sharp dip in the disease prevalence.

P-10 strategy produced the optimal efficiency at low disease prevalence (0–5%). In this study, the P-5 strategy was found more efficient as compared to the P-10 strategy as 19.31% more samples were tested during P-5 as compared to the P-10 strategy during the period. An increase in test specimens from P-5 to P-10 was not observed due to a smaller number of samples received associated with a very low prevalence of SARS CoV-2. Therefore, the P-10 strategy could not be explored for maximal testing efficiency. However, it was more effective in saving time, money, valuable resources, and manpower.

Appropriate regional pooling strategies based on the low incidence of the disease in diagnostic facilities could be a game-changer in covering a larger sample size including symptomatic, asymptomatic, and close contacts patients in large numbers. This proved to be a successful step as even during high lab positivity we were able to identify low disease prevalence areas, this regional analysis for positivity rate proved to be an effective measure to scaleup of diagnostic services in public facilities to curb the ongoing COVID-19 pandemic. The pooling intervention has been observed as a resource-saving approach that plays an important role in saving PCR reagents, nucleic acid extraction kits, and consumables including plastic wares. Time taken for testing was also preserved thus effectively contributing to enhancing the throughput of diagnosis. The regional pooling interventions showed less occupancy of manpower and instruments (automated RNA extractors and qPCR machines).

Therefore, it presented a proof of concept especially for resource-limited countries and at a time when test kits were short in supply. Adapting the pooling strategy helps to maximize resource-saving under a fluctuating prevalence rate. Also, the fraction of positive samples tested in pools can vary over time depending upon multiple factors that are changes in public health modified methods for example lockdown, closing of school, and social distancing. By P-5 and P-10 testing, we saved a significant number of test kits, manpower, time, and other resources as well as effectively identified the infected SARS-CoV-2 individuals in the population rapidly.

Similar cost-effectiveness of consumables in pooling of 5 and 10 samples have been reported from India based on a small sample size (Prakash et al., 2021). Whereas pooling of 4 samples resulted in the preservation of 66% of consumables (Singh et al., 2021). Savings of resources was found to be 36% and the turnaround time was reduced by 30%. Taken together from the this study and previous reports it is evident that the pooling of samples may decrease the assay sensitivity from 99 to 81% when compared with individual sample testing (Bateman et al., 2021). The efficiency of pooling depends strongly on the prevalence of infection and the optimal selection of pool size (Sawicki et al., 2021).

The average Ct value difference in low viral load samples (Ct ≥ 30) 2.48 and 3.05 in P-5 and P-10, respectively, while in high viral load (Ct < 30) 2.09 and 2.78 in P-5 and P-10, respectively which is similar to the previous findings from India and other countries (Abdalhamid et al., 2020; Farfan et al., 2020; Mahmoud et al., 2021). A twofold dilution of the positive specimens can increase the Ct value by 1.24 (Wacharapluesadee et al., 2020; Yelin et al., 2020). In P-10 the overall average Ct value is observed between pools and unpool sample is 2.84 which slightly varies from the previous finding of the 10-pool strategy (Praharaj et al., 2020). This might be due to the large sample size, and the difference in kits used for RNA extraction and RT-qPCR (Garg A. et al., 2021). Other factors may also affect the sensitivity of RT-qPCR, like the sample collection technique, type of samples (NPS, oropharyngeal, nasal, etc.), the sample size in the study, cold-chain maintenance during transportation, viral load in the sample, and regional positivity (Afzal, 2020; Arevalo-Rodriguez et al., 2020). The rate of individual sample testing was high and false-negative results are low in both pooling strategies. However, in specimens with low viral load (Ct ≥ 30), the negative results in flagged pools (amplification of either screening or confirmatory viral targets in pools) are more as compared to the high viral load (Ct < 30) in P-5 and P-10. Contrary to the current observations, lower percent positive or non-positive in specimens with higher *C*_*t*_ values and higher percent positive with lower *C*_*t*_ values were also reported earlier (Bateman et al., 2021). Samples with borderline Ct values when diluted to 1:5 or 1:10, will show a Ct value more than the pooled positive Ct value and reported as negative in RT-PCR. In another study, authors have also performed pooling of extracted RNA samples and observed higher Ct values as compared to pools (Abdalhamid et al., 2020). There are certain limitations of the pool testing which shall be considered on priority. Some of the borderline positive samples (low viral loads) are most likely to be missed because of further dilution of viral targets with negative samples and degradation in repeat freeze-thaw during repeat test while in unpool. The limitation of the pooling in the negative results is as the pools that declared negative may have individual samples which were inconclusive/invalid. The absence of amplification of Internal Control genes can be due to improper sample collection, degradation of RNA due to improper storage or heat inactivation and expired VTMs, etc. The heat-inactivation and pooling efficiency does not affect the result (unpublished results). Individual samples from negative pools shall also be tested to calculate the efficiency of the pooling strategy.

The pooling strategies when performed in settings with low to moderate COVID-19 prevalence have been effective in resource-saving and efficient in high-throughput. Effect of pooling approach which can facilitate mass screening in early days of disease prevalence, and diagnosis of suspected cases based on regional positivity.

## Data Availability Statement

The original contributions presented in the study are included in the article/supplementary material, further inquiries can be directed to the corresponding author/s.

## Ethics Statement

The studies involving human participants were reviewed and approved by Institutional Ethics Committee (IEC). Written informed consent for participation was not required for this study in accordance with the national legislation and the institutional requirements.

## Author Contributions

PK and SS conceptualized the study. PM, MS, SP, NeC, NiC, AM, DS, RS, NS, and SM performed the experiments. PM, MS, SP, NeC, NiC, AM, DS, RS, NS SM, AaK, and NN compiled the data. PK, AG, AnK, RSK, and SS analyzed the data. PM, MS, SP, NeC, NiC, AM, DS, AnK, and PK wrote the manuscript. All authors contributed to the article and approved the submitted version.

## Conflict of Interest

The authors declare that the research was conducted in the absence of any commercial or financial relationships that could be construed as a potential conflict of interest.

## Publisher’s Note

All claims expressed in this article are solely those of the authors and do not necessarily represent those of their affiliated organizations, or those of the publisher, the editors and the reviewers. Any product that may be evaluated in this article, or claim that may be made by its manufacturer, is not guaranteed or endorsed by the publisher.
